# Supplementary material for: Picea wilsonii NAC31 and DREB2A Cooperatively Activate ERD1 to Modulate Drought Resistance in Transgenic Arabidopsis
Source: Int J Mol Sci. 2024 Feb 7;25(4):2037. doi: 10.3390/ijms25042037 (PMC10888420; doi:10.3390/ijms25042037)
Supplement: Supplementary file 1 [file ijms-25-02037-s001.zip › ijms-2798998-supplementary.pdf]

Supplementary materials

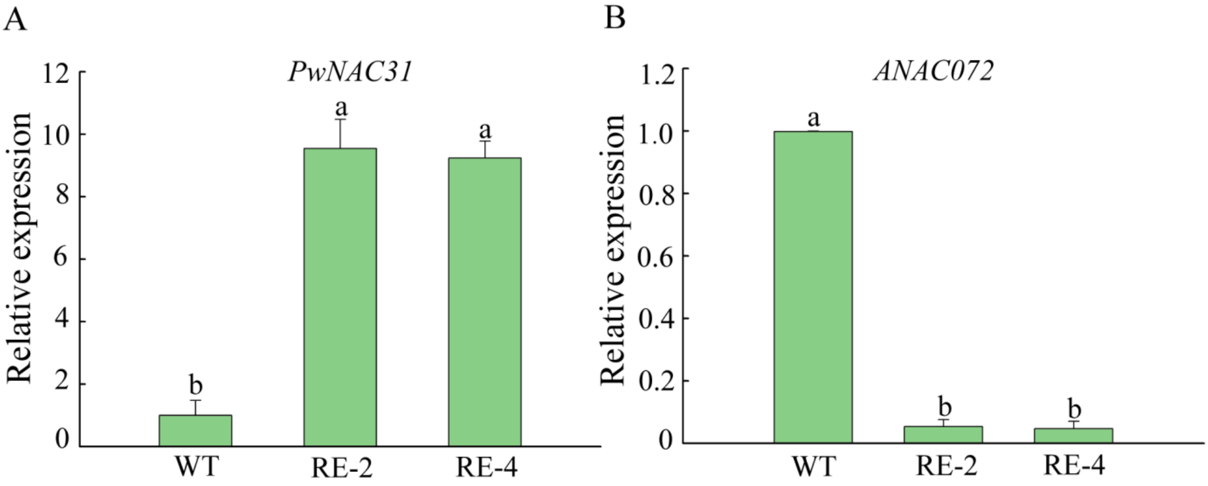

Figure S1: Identification of mutant and restore lines in *Arabidopsis.* (A) The expression quantity of *PwNAC31* were examined in T3 transgenic lines and WT *Arabidopsis* seedlings. (B) The expression quantity of *ANAC072* were examined in T3 transgenic lines and WT *Arabidopsis* seedlings.

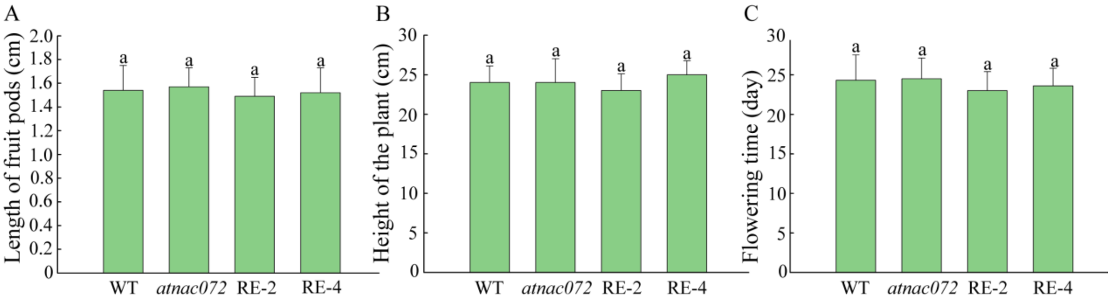

Figure S2: The growth condition of mutant and restore lines in *Arabidopsis*. (A) The fruit pods length and (B) plant height of 4-week-old transgenic and wild-type lines were measured under normal growth conditions. (C) Flowering time, examined as number of days required for flowering**.**

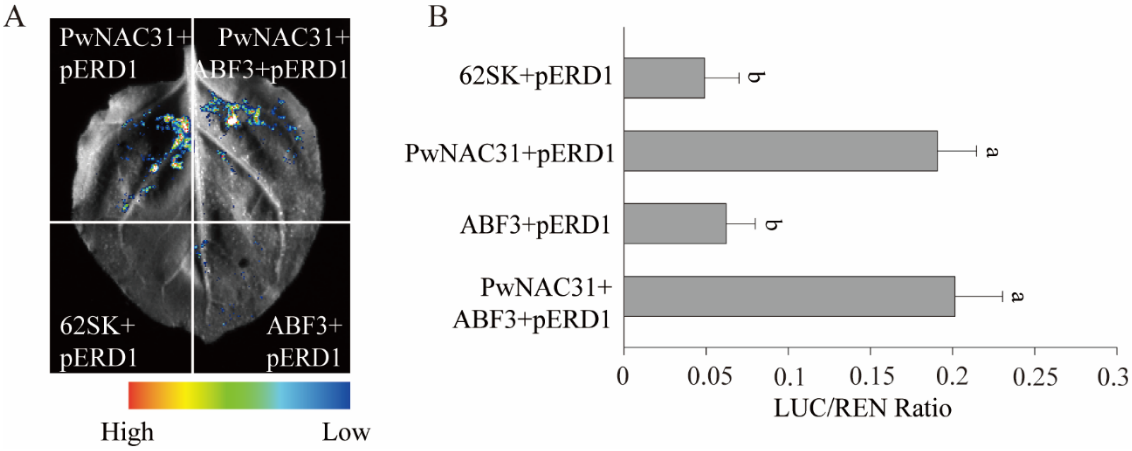

Figure S3: Dual-luciferase assay of ABF3 dose not activate the *ERD1* promoter. The left is dual-luciferase imaging, and the right is dual-luciferase activity assay. The error bars indicate (±SD) of three independent replications and the different lowercase letters in Figure D mean significant differences at 5% level treatments.

Supplementary Table S1. The CDS sequences used in this study.

Gene Name	CDS
PwNAC31	TTGGTACATACATGTATGCGCGAGATTGGAAGAATGGGCTGCAAATCAGGACCAAACTCTGCGCAGAT<br>CAATCTGCCCCCAGGTTTCAGGTTCTATCCCACGGACGAGGAATTGATGGTGCATTACTTGTGTAAAAG<br>AGTGGCTTCACAGCCCTTTCCAATTCCGATTATTGCAGAGATTGATTTGTACAAGTTCGATCCGTGGCA<br>ACTACCCGAGAAGGCGTTATTTGGGGAGAAAGAATGGTACTTTTTCAGTCCACGTGACAGAAAATACC<br>CAAATGGGTCGCGTCCCACCGAGCTGCGGGATCTGGGTATTGGAAGGCTACTGGGACGGACAAACCT<br>GTTACTGCTGGACTGAACAGGATGAAGGTCGGGGTGAAAAAGGCTTTGGTTTTCTACAAGGGGAAG<br>GCTCCCAGAGGTGTCAAGACCAATTGGATAATGCATGAATATCGTCTTGCAGACAGTGCCAGCAGGGC<br>TGCAAAGAGAAAAGGAAGCATCAGGTTGGACGACTGGGTACTGTGTCGAATATATGAAAAGCACTAC<br>GCCAGTCAGAAGGCCGTGCCTAAAGAAAGCAATGAGTTATCATCATGCGTAGATCCAGAAGAAGATCA<br>ATATATGGCACCCGTTGCATCGAATTCCCCTCCAGACATGGACGATAACAAGCTGCTCATCAATCTGCCG<br>CGAATCAATTCCCTGAACAGAATAAACTCTCTTCTGAAATTCCCCAGCAGGGAGATCATGAAGATGCCA<br>GACCCGAACTACAACCAGACGGAGCAACAATATTCCCGTGCAGAAACAGATGCCGGTTCTAATTCCGG<br>AGGCCAGGCGATAGAATATGGTCAGAATTCACCGTTCTGGAGAAACCAGCAATTCGGATACAACTATG<br>ATCAGATGGCGCAGAACCCGTTTCCTCAACAGTTAGAGCATATGCCATCCTTGGGAGCCAATTCTAGTG<br>CGTTTTTTGCAAGCCTGGCTGCAGAGCTCCTTTAG
DREB2A	ATGGCAGTTTATGATCAGAGTGGAGATAGAAACAGAACACAAATTGATACATCGAGGAAAAGGA<br>AATCTAGAAGTAGAGGTGACGGTACTACTGTGGCTGAGAGATTAAAGAGATGGAAAGAGTATAA<br>CGAGACCGTAGAAGAAGTTTCTACCAAGAAGAGGAAAGTACCTGCGAAAGGGTCGAAGAAGGG<br>TTGTATGAAAGGTAAAGGAGGACCAGAGAATAGCCGATGTAGTTTCAGAGGAGTTAGGCAAAGG<br>ATTTGGGGTAAATGGGTTGCTGAGATCAGAGAGCCTAATCGAGGTAGCAGGCTTTGGCTTGGTAC<br>TTTCCCTACTGCTCAAGAAGCTGCTTCTGCTTATGATGAGGCTGCTAAAGCTATGTATGGTCCTTT<br>GGCTCGTCTTAATTTCCCTCGGTCTGATGCGTCTGAGGTTACGAGTACCTCAAGTCAGTCTGAGG<br>TGTGTACTGTTGAGACTCCTGGTTGTGTTCATGTGAAAACAGAGGATCCAGATTGTGAATCTAAA<br>CCCTTCTCCGGTGGAGTGGAGCCGATGTATTGTCTGGAGAATGGTGCGGAAGAGATGAAGAGAG<br>GTGTTAAAGCGGATAAGCATTGGCTGAGCGAGTTTGAACATAACTATTGGAGTGATATTCTGAAA<br>GAGAAAGAGAAACAGAAGGAGCAAGGGATTGTAGAAACCTGTCAGCAACAACAGCAGGATTCG<br>CTATCTGTTGCAGACTATGGTTGGCCCAATGATGTGGATCAGAGTCACTTGGATTCTTCAGACATG<br>TTTGATGTCGATGAGCTTCTACGTGACCTAAATGGCGACGATGTGTTTGCAGGCTTAAATCAGGA<br>CCGGTACCCGGGGAACAGTGTTGCCAACGGTTCATACAGGCCCGAGAGTCAACAAAGTGGTTTT<br>GATCCGCTACAAAGCCTCAACTACGGAATACCTCCGTTTCAGCTCGAGGGAAAGGATGGTAATGG<br>ATTCTTCGACGACTTGAGTTACTTGGATCTGGAGAACTAA
ERD1	ATGGAGGTGTTATCTACTTCCTCACCTCTAACGCTTCACTCCCACCGACTCCTCTCTGCTTCTTCTT<br>CTTCTTCACATGTCACCTCCATCGCCGCTTCTTCACTTTCTTCATTCGCTTCTTCATATCTCGGAAT<br>CTCCCTTTCTAACCGCACGATCCACCGCTTCTCCACAACTCCGACGAACTTAAGACGATTTCCTC<br>AAAGAAAACGGAAGAAATTCACTCCGATTTCAGCGGTTTTCGAACGGTTCACCGAACGAGCGAT<br>CAGAGCTATAATCTTCTCTCAGAAGGAAGCTAAATCGTTGGGAAAAGATATGGTTTACACGCAGC<br>ATCTTCTCTTAGGTTTGATCGCTGAGGATCGTGATCCTCAAGGCTTCCTTGGATCCGGTATCACCA<br>TCGACAAGGCTCGTGAAGCTGTTTGGAGTATTTGGGACGAAGCTAATTCCGATTCGAAACAGGA<br>GGAAGCTTCTTCTACTTCGTATTCAAAATCGACGGACATGCCTTTCTCTATCAGCACGAAACGAGT<br>CTTTGAAGCTGCGGTTGAGTACTCTAGGACTATGGATTGTCAATATATCGCACCGGAGCATATAGC<br>CGTTGGACTCTTCACCGTTGATGATGGTAGCGCCGGCAGAGTTCTCAAGAGATTGGGAGCAAATA

	TGAATTTGCTCACAGCTGCAGCACTTACAAGACTTAAAGGAGAGATTGCTAAAGATGGAAGAGA<br>GCCATCTTCTTCATCTAAAGGGAGCTTCGAGTCTCCTCCTAGTGGTCGAATTGCTGGTTCTGGACC<br>TGGTGGAAAAAAAGCGAAAAATGTACTGGAACAGTTCTGTGTGGATCTGACAGCACGTGCTAGT<br>GAGGGTCTTATTGATCCTGTTATTGGTCGGGAAAAAGAGGTTCAAAGAGTTATCCAGATACTTTG<br>CCGCAGAACTAAAAACAACCCAATTCTTCTTGGTGAAGCTGGTGTTGGGAAGACTGCCATTGCT<br>GAAGGACTAGCGATTAGTATTGCAGAAGCCAGTGCTCCTGGATTTCTCTTGACGAAACGCATCAT<br>GTCCCTGGATATAGGATTGCTAATGGCCGGTGCAAAAGAAAGGGGAGAGCTGGAGGCTCGAGTC<br>ACTGCTTTGATAAGCGAGGTTAAAAAATCAGGTAAGGTCATTCTCTTCATAGATGAAGTGCATACA<br>CTTATTGGGTCTGGCACAGTTGGAAGAGGGAACAAAGGGTCTGGGCTTGACATTGCTAACCTCTT<br>GAAACCATCACTTGGAAGGGGTGAACTTCAGTGCATTGCATCCACAACCCTTGACGAATTTAGGA<br>GTCAGTTTGAGAAGGACAAAGCCCTTGCAAGGAGATTTCAGCCAGTGTTGATTAACGAGCCAAG<br>CGAGGAAGATGCGGTGAAGATTTTGTTGGGCCTTCGTGAAAAATATGAAGCCCATCACAATTGCA<br>AATATACTATGGAAGCCATAGATGCTGCAGTGTACCTTTCATCACGATATATCGCTGATAGATTTCT<br>TCCAGATAAAGCTATTGATCTCATTGACGAGGCAGGAAGCAGAGCTCGTATTGAAGCTTTTAGGA<br>AGAAAAAGGAGGATGCAATCTGTATCCTATCGAAGCCACCTAATGATTACTGGCAAGAGATCAAA<br>ACAGTTCAGGCCATGCACGAAGTGGTTCTCTCAAGCAGGCAAAAGCAGGATGACGGTGATGCCA<br>TTTCAGATGAGTCTGGTGAACTAGTTGAAGAGTCTTCTTTGCCACCTGCAGCAGGCGACGATGAG<br>CCTATACTGGTGGGACCTGATGATATTGCAGCCGTTGCATCGGTTTGGTCTGGAATTCCAGTTCAG<br>CAGATCACTGCAGATGAAAGAATGCTTCTTATGAGTCTAGAAGATCAGCTTAGAGGCAGAGTTGT<br>TGGTCAAGATGAGGCGGTAGCTGCCATATCTAGAGCTGTGAAGAGGTCCCGGGTTGGCTTAAAA<br>GATCCCGACCGTCCAATTGCTGCTATGCTTTTCTGTGGACCAACAGGGGTTGGAAAAACGGAGCT<br>TACAAAAGCTCTGGCAGCAAATTATTTTGGCTCGGAAGAATCCATGCTGAGATTGGACATGAGTG<br>AATACATGGAGCGTCATACTGTGAGCAAGCTGATAGGCTCTCCTCCTGGTTATGTTGGCTTTGAAG<br>AAGGTGGAATGCTTACAGAAGCTATCAGGAGACGTCCTTTTACAGTGGTTTTGTTCGATGAAATA<br>GAGAAAGCACATCCGGATATCTTCAATATTCTTCTCCAGCTGTTCGAAGATGGCCATCTAACTGAT<br>TCACAGGGAAGGAGAGTATCTTTTAAGAACGCATTGATCATAATGACCTCTAATGTCGGATCATTG<br>GCCATTGCAAAGGGAAGACATGGTTCAATAGGGTTTATCCTCGATGATGATGAAGAGGCAGCATC<br>TTATACCGGAATGAAAGCTTTGGTAGTCGAAGAACTCAAGAACTATTTCCGTCCAGAGTTGTTGA<br>ACCGAATAGACGAAATCGTCATTTTCCGACAGCTAGAGAAGGCTCAGATGATGGAGATCTTGAAC<br>CTGATGCTACAAGACTTGAAGTCGAGGCTCGTGGCACTCGGAGTTGGTTTAGAGGTGTCTGAAC<br>CCGTCAAGGAGCTCATATGCAAACAAGGCTATGATCCAGCTTACGGTGCACGACCACTCCGGAG<br>AACTGTCACAGAGATTGTTGAAGATCCACTCAGTGAAGCCTTTCTTGCCGGGAGCTTCAAGCCTG<br>GTGATACGGCTTTTGTAGTTCTTGATGATACCGGAAACCCATCGGTTCGGACCAAACCAGATTCTT<br>CCACTATACGAGTTACAGACAAAACATCGATCGCATAG
ABF3	ATGGGGTCTAGATTAAACTTCAAGAGCTTTGTTGATGGTGTGAGTGAGCAGCAGCCAACGGTGG<br>GGACTAGTCTTCCATTGACTAGGCAGAACTCTGTGTTCTCGTTAACCTTTGATGAGTTTCAGAACT<br>CATGGGGTGGTGGAATTGGGAAAGATTTTGGGTCTATGAACATGGATGAGCTCTTGAAGAACATT<br>TGGACTGCAGAGGAAAGTCATTCAATGATGGGAAACAATACCAGTTACACCAACATCAGCAATG<br>GTAATAGTGGAAACACTGTTATTAACGGCGGTGGTAACAACATTGGTGGGTTAGCTGTTGGTGTG<br>GGAGGAGAAAGTGGTGGTTTTTTCACTGGTGGGAGTTTGCAGAGACAAGGTTCACTTACCTTGC<br>CTCGGACGATTAGTCAGAAAAGGGTTGATGATGTCTGGAAGGAGCTGATGAAGGAGGATGACAT<br>TGGAAATGGTGTTGTTAATGGTGGGACAAGCGGAATTCCGCAGAGGCAACAAACGCTGGGAGA<br>GATGACTTTGGAGGAGTTTTTGGTCAGGGCTGGTGTGGTTAGGGAAGAACCTCAACCGGTGGAG<br>AGTGTAACTAACTTCAATGGCGGATTCTATGGATTTGGCAGTAATGGAGGTCTTGGGACAGCTAGT

	AATGGGTTTGTTGCAAACCAACCTCAAGATTTGTCAGGAAATGGAGTAGCGGTGAGACAGGATC<br>TGCTGACTGCTCAAACTCAGCCACTACAGATGCAGCAGCCACAGATGGTGCAGCAGCCACAGAT<br>GGTGCAGCAGCCGCAACAACTGATACAGACGCAGGAGAGGCCTTTTCCCAAACAGACCACTATA<br>GCATTTTCCAACACTGTTGATGTGGTTAACCGTTCTCAACCTGCAACACAGTGCCAGGAAGTGAA<br>GCCTTCAATACTTGGAATTCATAACCATCCTATGAACAACAATCTACTGCAAGCTGTCGATTTTAA<br>AACAGGAGTAACGGTTGCAGCAGTATCTCCTGGAAGCCAGATGTCACCTGATCTGACTCCAAAG<br>AGCGCCCTGGATGCATCTTTGTCCCCTGTTCCTTACATGTTTGGGCGAGTGAGAAAAACAGGTGC<br>AGTTCTGGAGAAAGTGATTGAGAGAAGGCAAAAAAGGATGATAAAGAATAGGGAATCAGCTGC<br>AAGATCCCGCGCTCGCAAGCAAGCTTATACGATGGAACTGGAAGCAGAAATTGCGCAACTCAAA<br>GAATTGAATGAAGAGTTGCAGAAGAAACAAGTTGAAATCATGGAAAAGCAGAAAAATCAGCTTC<br>TGGAGCCTCTGCGCCAGCCATGGGGAATGGGATGCAAAAGGCAATGCTTGCGAAGGACATTGAC<br>GGGTCCCTGGTAG

Supplementary Table S2. The promoter sequences used in the study.

Gene Name	Promoter
PwNAC31	GTGTGGAATTGTGAGCGGATAACAATTTCACACAGGAAACAGCTATGACCATGATTACGCCAAGC<br>TTGCATGCCGGTAGAGGTGTGGTCAATAAGAGCGACCTCATGCTATACCTGAGAAAGCAACCTGA<br>CCTACAGGAAAGAGTTACTCAAGAATAAGAATTTTCGTTTTAAAACCTAAGAGTCACTTTAAAAT<br>TTGTATACACTTATTTTTTTTATWACTTATTTAATAATAAAAATCATAAATCATAAGAAATTCGCCCG<br>GAATTAGCTTGGCTGCAAGCGCGCAAAAAACCCCTCAAGACCCGTTTAGAGGCCCCAAGGGGTT<br>ATGCTAGTTATGCGGCCGCTGCAGGTCGACGGATCCGCCTGATGCTTCCTTTTCTCTTTGCAGCCC<br>TGCTGGCACTGTCTGCAAGACGATATTCATGCATTATCCAATTGGTCTTGACACCTCTGGGAGCCT<br>TCCCCTTGTAGAAAACCAAAGCCTTTTTCACCCCGACCTTCATCCTGTTCAGTCCAGCAGTAACA<br>GGTTTGTCCGTCCCAGTAGCCTTCCAATACCCAGATCCCGCAGCTCGGTTGGGACGCGACCCATT<br>TGGGTATTTTCTGTCACGTGGACTGAAAAAGTACCATTCTTTCTCCCCAAATAACGCCTTCTCGGG<br>TAGTTGCCACGGATCGAACTTGTACAAATCAATCTCTGCAATAATCGGAATTGGAAAGGGCTGTG<br>AAGCCACTCTTTTACACAAGTAATGCACCATCAATTCCTCGTCCGTGGGATAGAACCTGAAACCT<br>GGGGGCAGATTGATCTGCGCAGAGTTTGGTCCTGATTTGCAGCCCATTCTTCCAATCTCGCGCATT<br>GCCATGGCCATATGCAGGTCCTCCTCTGAGATCAGCTYSYKCTCCTCCATGATGGCGGCTCGCCCT<br>ATAGTGAGTCGTATTACAAATTCCGGCGATACAGTCAACTGTCTTTGACCTTTGTTACTACTCTCTT<br>CCGATGATGATGTCGCACTTATTCTATGCTGTCTCAATGTTAGAGGCATATCAGTCTCCACTGAAG<br>CCAATCTATCTGTGACGGCATCTTTATTCACATTATCTTGTACAAATAATCCTGTTAAC
DREB2A	ACAGAGAAGCAGGCTCCCATCTCTCATAGATTCTCAAATTCCTACCAGAGAGATTAATATACAAATTCCT<br>TTCCTAATGTTTTAGTTTATTAACTGAGGTGTGTGCACGATCATTCGGGTTGACCACCAATCTTGGCATC<br>CAGTCATCTACGACTCGATCAAATATTTATGTTATTTTAAAAATGAACTGTAACCAAGATAACATTTTTTT<br>CATAAGGTCAACGACCCTTGGGATGTTAACAGAGCCAAGTTTAGTGAGCACTATGTGCTCGTTTTTTCT<br>TAACACAATTCTAACGTGGTTTGGCCAAGCCAAGTCAACATTTTACTTACATCTCGTTTTCCAATAATGG<br>TTTTGTAATCGATAACCTAAATCATTGTAATGAATGCGTTCCTCCCTATCGATCCTAGGCCTTAGACAATG<br>CTGAATGATTCATAGCCACGCGAATAACCTATTCATACTAATATGGAAAGAAAGAAGCCAAACTTACAG<br>AGCTCTTCTCACGGTCGTCGAAGAAAAGAGAGTCTACAGTCAGCAACAAAATTAGTGTTGCCATCATG

	GCATCATCTTGCAGCTTTTTCCGCACAAATAATTTATCATCCAAATGTTGTTCACTAAACCAAAAACAAA<br>CAAGCATTACAGCGAAGAAACCAACTCGTAGATACAGATTTCCAAATTCATGCCTTATTTAGACCAATAA<br>AAACTGAAATTTCTCTTCAGCGAAAAAAAAAAACAAACAAACTGAAATTTCATATAGATCCAGAAGATAG<br>AAACTTGTAGGCTCAATCGACTAGACTAGAAGATGCTCACCCGATCGTGCTTGAGATAGCGAGATAGTA<br>GCAACACCGACGGTAGAAATAAAATGGACGACACCCATCCAATGGGCTAATTTAGATTAACGGGCTTT<br>AAGGGTTTGATAATGGATGTTAATTAACTGAGGCACATGGGATTGTATCACGTAGGCAATGGGTTTGAT<br>AATGGATGTTAAGTAACTAAGGCCCATGAGGTTGAAGTACGTAGGCAATTGCGTGAGCTTACGTTAGC<br>GATGCCGTTAGAGACACGTAGTGGATGAAGTGGCTTTGGTTAGCAAAGGACACATGAGGCACATGCA<br>AAGGCTATAAATGACTGCTGCTTTGCTACAACTTGCGATTCCCAAATTTTATAAGGTAATGGACCCTATC<br>ACCTCTGCTCGAAGCTAAGCCACCCAAGTTTGAGCTTCACCATTTGACACGTCTCTAGCTAATACTTAAT<br>GCTTAAACTTTAAACTAATACTTCATGTTTAAGACATTTCTGGCTGACACATTTATGAATTCGCTCTATGT<br>CGTACGTACACCGGAAACCTTTAATTTTACAATATTAACCGTGATCTTTTTAAAAATATCTATATAAACGAT<br>AAGTATCTCATCAAAACAAAATTGAATAATGTGCACGTTTGTAAAAACTAGAAAAACAGGCAATAAACA<br>TCATCATCCAATTACACATCTAGTAAGTGTGATGCAGTGGCAAACTGGCAATAAACCAAGAAAAATCGA<br>GAAAGAGCAGATGAGACAGTGTTGTGTTGTCAGGGTTAATAAAAAAAAAATGAAGATATTTTAAAATT<br>TCATAATATTTAAATAATGAAGTAGTTTTATGATCTTATCCATAAATCAATTTTAAAAAGGTTTAAACTTTA<br>CTTTTCCGTATCAACAGCGTGTTTCGAGAAGATTCGGGAGGACACTCGTCGAACGGAAAAGTCGTCTA<br>AGCCTTTATGTTCGAATCAAAAACTGACACGTAACCTTGCTCTCAAACAGAAAAATAAAATAATGTTAG<br>AAAAATCTAGAGAAGGCTATAAATACTCCGTAGATACTTTGTCTTCCTT
ERD1	TGGTCTTATGGAATCTCTCACTACTTTTTTAGAGGAAAATTTTGTAAACGCAGAGACAACATTTTTCCCG<br>AGAAAATGAGAGAATTTTTCCGGGAAAATATTTGATGTTATTCTTCTCTCTGAAAGTAAACGATCTCTCA<br>AGTAATTTTTGATAGGAGAATGAGAAAATGGATTCGTATAAATAGTGAAGAGAAGAGACGAGTAAAAG<br>AATTTGAATAAAGAAGAGAGACGTGGTCGGACTTGGCCCACCAACTTTGTCATCTATGATGCAAAATAT<br>TTAACAAATAACAACTAATTGAGGTTGTAACGTGCATTTGTAATGCAATACATTGAGAAAAAAACAAATA<br>TTATACATAACAAAGTTGAGTTTTTTTCACTAGTTGTTTATTTAAGAATTAGAACAATAGTCCACTGTTTT<br>TTAACAAGTCCATAGCGAATGTTGGAAACTTATTTTAAAAAATTATTAATTGGAAGTCTAAAGAATTACG<br>AAAACAAATTGTATTTGATTAAAACAAAGTACAAATTGCATTTAAAAAGAAATAGTCTATAGAACATGGA<br>TGATCCTTCCATATAAAGTTTGATCATGCACATAGTATGACAATAATCAAAGAGCGGGGATAGCTGTGAA<br>TCTTTAGCAGCGAAAACTTCAAACATATATTTCATGAATACAACAATGTTATAAAATGAATTTCGTACGAC<br>ATAATAAATGTTATAGTAATTCTTATGTTTGTCCAAAAAAAAGAAAGTAATTCTTAGGTTAAATATATAAA<br>AACATGACAAAAGTGTAACTATTAAAGATATTTGATGTACTACATATATATAACAAATTTTATCAAAGATGT<br>ACATAGACAACTATGTTCTTAAAACTCCACAAACATTACGGCAGTTAGAATTTAAATTTATCTTGGAATAT<br>TGTTTGTATATTATTAACAAATGTAAATTGGTGTAGGTTTTCAGATTTTCATAATGAAAACAGTTTTAAATT<br>ATGGTTACGAGATAATTTTGACGAATATGAATACATATATAGAAAATGTTTGGGCCTATATCTATGAAAAT<br>GTTTGGGCCTATCTATATCTATGAAAATTGTATGAGATACCAACAAAATAAAATATTTAAAGTAGCAAAAC<br>ATTGTGTTACTTTTCTATTATCTTGTCTTAAATAAATAAAAATCAATTTTTTTTTTAAGAAGTAACACAATG<br>TGAAGAATACGTGTGTACTTTTTTTTTTTTGGCGTAAATAAAATTAGTAAATTAAGACAAGATTAATAGA<br>AAAGTACACACGTATTCTTCCCATTGTACGTTAAAAAGCTCGTGAATTCACATGCATGAGGACGATTTG<br>GGTAACCAATAAGTTGACTATATTTTTGTATGTCTTTCTATTTTTCTTCACATGGAAAGAATTTTCCAACG<br>AAAAGTTGAAGGGGAGACAATTAGGAACACTAAATTGTCACCATTAGGCTCTTAAGTTTTAATTTGGC<br>AACCAATTTCATTTTCTCTATTCTGTATTTTTGGATTGGGACCGTTTTATTTGTTTGTGTGTGTGCAACTT<br>GAGCTCTTCTTCTGTAACACGCATGTGTTGCGTTTGGTTTTTAATTTATTTGCCTACTTTTGCGTTTTCAA<br>CGTTTGTTACCACATTTGAACTATAAAGTCCAATTCCATACCGATCCTCTACAACCATATAAATGTGCTAC<br>AACAAAAGTTAATCTTTGATCTTGATCAACTCAAAGATGACTATACATGATTAGTTCTCCTAAAAGCTAAT

	GTTGTAGAGTGCAATTGCCGACAAACCTCCAAGTTTTGTGTCAACTTGAATTTGTA
ABF3	AAACTCTCTCTTTTTTGTTAAAAAAAGGTTATATATCTTTTAACCTTCTAGGATTTTAGCCTAGCTGAAAA<br>AAAGTTACACATTAAACTTGTTTTTGTTAACAACGTTTTAGCATTTCGGTTGTGTGTTGTTTCTTCCGGT<br>TGTTATAGTTTCGTACTTTCGTTGGTTTTGAGTTTTGGATTCTTGAAAGGTGGTTATAATTTATACATTTT<br>GTTTCTACACGACCTTAAAGAAGTAACTTCAATTAATTAAATCTAATTCGGGAAAGAGCACGTCTCAAA<br>AGAGTCGTCGAGTCCCATGTGAATTCTATATGATAACATTAAACAAAATCACGGTTCCATATTTGTTTAAT<br>ACTAGTATGTATTGCAGAGTAGACTTTCCTAATCCAGTAGATTCTTAAAGAAAAAATAATATAAAAAGAT<br>TGAAAATAGAAATAAAAGATGGGAGACACGAAGGGTCACTTGTATTGTACCGAGTCCAAATGTAAAGA<br>AGTCTCAATAATGGTGTAATAAAATAAAGAAGTTGAAGACAAGATCACAATCACCATGTGAAGATCAAC<br>ACACAAGATCATAATTTTATTGTATGTTTGCTTCATTTCAACTTTTTTAAATATTGTTAAGAGGGCAGGCA<br>TTGAGTGCGCCACGTGGCAAATGACCATCCCTTTGTAGGTGCAGCAAACGTGATAAAGATTCCGGACT<br>GTTCACCCATTCCCTTCTTCCATTTGTTTCTTATTTTTTCTTACAAATCTTATCGTATCATAATTTTTTTTCCA<br>TAATGCGCTCCCTTGAATGGAATAACGTTGGTTATTTTGTTTCCAACCAAATTTTCATACCATTCGATCAA<br>ATAGGTGCATCAACTTACAAGAAATGAAAGTGTGATCCCTATTTTATTAGGTAGCTAAGTAAGTGTTAGT<br>GAACACTACGTTAGATTGTTACTCAAATAACGGATCAACGAATCTCGTATTCATTACTATACTGCTAATGA<br>ATGCATCAATTTTAATTTTTATTGGTTATCTATCCTTAAAAAAAAGTTATAAGAATATTTTGATAATTTGTTC<br>CAAGATGTAGTACTAGTAGTAGTACTTGGTCTCGTAAACGACGACGCCGTTTAGGAAATCAAACGGCC<br>AAAAATGCTTCCACTTTGCGAAATTTTATTGGTGGGAAACGCAAATGTATCGATAATGATTTAAAGAAA<br>AAGCGGCGTTTCTAATTGGACCACGTTGCCCAATCTCTACGGCTCACGCACGATCTGACGTCAAATTGG<br>CATAATACTATAATAACCTTATCGAGAATAAAATAATAGTCGCATCCAAACAAAAGAAACCAACCCTTTTA<br>TATCACGACGAGTGTCACGCCATGGACACTTGTCCATCTCTCTCGCTCTGACCCCTTTCAGGTATCTTTTA<br>TTTCTCAAAGAGGAATTATTGATTTTCCATTTCCAAAGAAAAAAAATAAATTCGAAGGTCAGGAAAATT<br>AACAAAAAACTTCCTTTTTTTTTTTGTTAGTTTGTGTGACTGAGCTGCTTCATTTTTTTTCTTTCTTTTTT<br>TTTTTGGTTTGATGAATCGATTTTTGTTGTCTATTACTGATTGGTTTTCTTGTTCAGATTCACTGATTCGA<br>AGAGAATCATGATTTTTTTTTCCCGCTGAATAATAAGCATATGATTGGGTGTTTTGGAGATTTGTTTACT<br>GATTAAAAGGAGATTCCTTTCCATTTTCACCATTTGCTCTGTTTGACTTCATTGTGCTTATATTTCATTTAG<br>ATCTTTTGTTTGGGTTTAGCTTTGGAACTGATAAAAATCTGATTTTGTCTCACGGCTTTGGATTTGGTTC<br>TTAAATTTTGGTACTTTAAAACTGGATAAAGATCAGTGCTTTTTTAGATTCTTCGTTTGTTGATGAATTTA<br>TGGATGTATGTATAATTAAACCATAATCTCTCTGCTTGTTTGTTTTCTTATAGGTAAATATCCAGAAGCTTG<br>ATCCTCCTAGTTGTACGAAAGCTTGAGTA

Supplementary Table S3. PCR primers used in this study

Gene Name	Gene No.		Sequences(5´to3´)
PwNAC31		Forward	TCCGAGCTTTTGTCTTTGTTT
		Reverse	AGAGCAGAGCAGAATGAATGA
ABF3	AT4G34000	Forward	GATGTGGTTAACCGTTCTCAAC
		Reverse	CAGCTTGCAGTAGATTGTTGTT
DREB2A	AT5G05410	Forward	CATGTTTGATGTCGATGAGCTT
		Reverse	ATTCCGTAGTTGAGGCTTTGTA
ERD1	AT5G51070	Forward	CTTTCTCTATCAGCACGAAACG

	Reverse	CGGTGCGATATATTGACAATCC
PwNAC31-pro	Forward	GTGTGGAATTGTGAGCGGAT
	Reverse	GTTAACAGGATTATTTGTACAA
DREB2A-pro	Forward	TTTCAACAAATCATCTCGTCTTG
	Reverse	CATCTCCTTCCCAGAAACAA
ERD1-pro	Forward	AGTGAAGAGAAGAGACGAGTAAAAGAA
	Reverse	GATCGAATTCGTCGTCGGT

Supplementary Table S4. Primers for vectors' construction in this study

Gene Name		Sequences(5´to3´)
ERD1pro-KpnI-pAbAi-F	Forward	TTCGAGCTCGGTACCAATTTGAATAAAGAAGAG
ERD1pro-SalI-pAbAi-R	Reverse	TGCCTCGAGGTCGACAGGAAGTAGATAACACCT
PwNAC31-NdeI-AD-F	Forward	GATTACGCTCATATGATGCGCGAGATTGGAA
PwNAC31-EcoRI-AD-R	Reverse	ACCCGGGTGGAATTCCTAAAGGAGCTCTGCA
DREB2A-AD-NdeI-F	Forward	GATTACGCTCATATGATGGCAGTTTATGAT
DREB2A-AD-EcoRI-R	Reverse	ACCCGGGTGGAATTCTTAGTTCTCCAGATC
ABF3-AD-NdeI-F	Forward	GATTACGCTCATATGATGGGGTCTAGATTA
ABF3-AD-EcoRI-R	Reverse	ACCCGGGTGGAATTCCTACCAGGGACCCGT
p0800-*ERD1*-KpnI-F	Forward	GGCGAATTGGGTACCAATTTGAATAAAGAAGAG
p0800-*ERD1*-SalI-R	Reverse	ATCGATACCGTCGACAGGAAGTAGATAACACCT
SK-*PwNAC31*-BamHI-F	Forward	AGAACTAGTGGATCCATGCGCGAGATTGGAA
SK-*PwNAC31*-SalI-R	Reverse	CCCCTCGAGGTCGACCTAAAGGAGCTCTGCA
SK-*DREB2A*-BamHI-F	Forward	AGAACTAGTGGATCCATGGCAGTTTATGAT
SK-*DREB2A*-SalI-R	Reverse	CCCCTCGAGGTCGACTTAGTTCTCCAGATC
SK-*ABF3*-BamHI-F	Forward	AGAACTAGTGGATCCATGGGGTCTAGATTA
SK-*ABF3*-SalI-R	Reverse	CCCCTCGAGGTCGACCTACCAGGGACCCGT
PwNAC31-AD-NdeI-F	Forward	GATTACGCTCATATGATGCGCGAGATTGGAA
PwNAC31-AD-EcoRI-R	Reverse	ACCCGGGTGGAATTCCTAAAGGAGCTCTGCA
DREB2A-BD-NdeI-F	Forward	GAGGACCTGCATATGATGGCAGTTTATGATCAG
DREB2A-BD-SalI-R	Reverse	CCGCTGCAGGTCGACTTAGTTCTCCAGATC
ABF3-NdeI-BD-F	Forward	GAGGACCTGCATATGATGGGGTCTAGATTAAAC

ABF3-SalI-BD-R	Reverse	CCGCTGCAGGTCGACCTACCAGGGACCCGTC
RHA2A-NdeI-BD-F	Forward	GAGGACCTGCATATGATGGGGCTACAAGGT
RHA2A-SalI-BD-R	Reverse	CCGCTGCAGGTCGACTCAGTGGAGAGAGAA
ZFHD-NdeI-BD-F	Forward	TAAAATGCGACACTACGTTCAC
ZFHD-SalI-BD-R	Reverse	CAGCTTTGTTGGAACATACCAA
PwNAC31-1300-F	Forward	CGCGGATCCATGCGCGAGATTGGAAGAATGGGC
PwNAC31-1300-R	Reverse	CGGGGTACCAAGGAGCTCTGCAGCCAGGCTTG
PwNAC31-1205-F	Forward	CCATCGATATGCGCGAGATTGGAAGAATG
PwNAC31-1205-R	Reverse	CGGGGTACCCTAAAGGAGCTCTGCAGCCA
PwNAC31-N-BD-F	Forward	CATGCCATGGCAATGCGCGAGATTGGAAGAATG
PwNAC31-N-BD-R	Reverse	CGCGGATCCGCCTGATGCTTCCTTTTCTCTTTGC
PwNAC31-C-BD-F	Forward	CATGCCATGGCAATCAGGTTGGACGACTGGGTACTG
PwNAC31-C-BD-R	Reverse	CGCGGATCCGCTAAAGGAGCTCTGCAGCCA
PwNAC31-BD-F	Forward	CCGGAATTCATGAGTTTATCAGTAAATGGG
PwNAC31-BD-R	Reverse	AACTGCAGATTACTTTACGAAGTTCCATAAATC
GFP-F	Forward	GTCCAACACGTCTCAAAGCAAG
GFP-R	Reverse	TCCATGCCGAGAGTGATCCC

Supplementary Table S5. qRT-PCR primers used in this study

Gene Name		Sequences(5´to3´)
PwNAC31-RT-F	Forward	CAACCAGACGGAGCAACAATATTC
PwNAC31-RT-R	Reverse	GTTCTGCGCCATCTGATCATAGTT
ABF3-RT-F	Forward	GATGTGGTTAACCGTTCTCAAC
ABF3-RT-R	Reverse	CAGCTTGCAGTAGATTGTTGTT
NCED3-RT-F	Forward	GATGAATTTGTTCCAGAGAGCG
NCED3-RT-R	Reverse	AACACTAGGATCAGCCGTTTTA
DREB2A-RT-F	Forward	CATGTTTGATGTCGATGAGCTT
DREB2A-RT-R	Reverse	ATTCCGTAGTTGAGGCTTTGTA
ABI5-RT-F	Forward	AATAAGAGAGGGATAGCGAACG
ABI5-RT-R	Reverse	GCTACCACCACCTCTATGTATC

RD29A-RT-F	Forward	TTCTGTAAGGACGACGTTTACA
RD29A-RT-R	Reverse	CGTACTCGTTACATCCTCTGTT
ERD1-RT-F	Forward	CTTTCTCTATCAGCACGAAACG
ERD1-RT-R	Reverse	CGGTGCGATATATTGACAATCC
PwEF1-RT-F	Forward	AACTGGAGAAGGAACCCAAG
PwEF1-RT-R	Reverse	AACGACCCAATGGAGGATAC
ATNAC072-RT-F	Forward	GATCCGTTAGCCCAGTTGAGT
ATNAC072-RT-R	Reverse	TAGCCTGCAACTTTCCGACAT
ACTIN-RT-F	Forward	GGTAACATTGTGCTCAGTGGTGG
ACTIN-RT-R	Reverse	AACGACCTTAATCTTCATGCTGC
